# Editorial: Improving post-surgical outcomes for patients with primary gastrointestinal stromal tumors

**DOI:** 10.3389/fonc.2023.1191322

**Published:** 2023-06-23

**Authors:** Leonidas G. Koniaris, Riad Haddad

**Affiliations:** ^1^Oregon Health and Science University, Portland, OR, United States; ^2^The Carmel Medical Center, Haifa, Isreal

**Keywords:** sarcoma, cancer, gastrointestinal, surgery, recurrence

For decades surgery was the main therapy to treat patients with gastrointestinal stromal tumors (GIST). Surgery provided palliation and the only chance for cure. In the 2000s the benefits of chemotherapy remained unclear. With the discovery that GIST expressed type III tyrosine kinase receptors effective molecular targeted chemotherapy became a reality (TKI). Outcomes for patients with GIST have dramatically improved since that time and resulted in many instances of improved palliation, prolonged survival and increased cure (1, 2). In this Research Topic “ *Improving Post-Surgical Outcomes for Patients with Primary Gastrointestinal Stromal Tumors*”, we explore topics in understanding the interplay of surgical and other ablative approaches with TKI therapies.

In, “Primary Tumor Resection Improves Survival in Patients With Metastatic Gastrointestinal Stromal Tumors: A Preliminary Population-Based Analysis” Zhao et al. from Nanjing Medical University examine a large cohort of 455 patients with metastatic GIST and observe a clear survival benefit for those provided surgical resection for the primary malignancy, particularly in the subgroup with 5-10 cm primary tumors and those with a tumor arising from the stomach.

In “Surgical Resection Is Still Better Than Endoscopic Resection for Patients With 2-5 cm Gastric Gastrointestinal Stromal Tumors: A Propensity Score Matching Analysis” Wu et al. from Shangdon First Medical University, Cheeloo University, and Peking University add to the results of Zhao et al. and support that less invasive endoscopic resection may be equivalent to surgery in carefully selected, 2-3 cm, good prognosis tumors based upon a propensity-matched 282 patient cohort.

The third paper of this series is, “Novel Prognostic Nomogram for Recurrence-Free Survival of Patients With Primary Gastrointestinal Stromal Tumors After Surgical Resection: Combination of Prognostic Nutritional Index and Basic Variables” This paper by Li et al. at Tianjin University Hospital reports a novel, clinically useful nomogram in monitoring and determining the need for adjuvant TKI therapy.

The final paper in this series, “Changes in imatinib plasma trough level during long-term treatment in patients with intermediate- or high-risk gastrointestinal stromal tumors: Relationship between covariates and imatinib plasma trough level.” The paper by Wu et al. from Chongqing University examined changes in imatinib plasma troughs during duration of therapy in a high recurrence-risk cohort.

Taking these four special manuscripts together, current data supports a primary role for margin negative resection for GIST as well as a palliative therapy in the setting of metastatic disease. Data supports endoscopic approaches for small and low-risk tumors if a margin-negative resection can be obtained. TKI therapy is a critical adjuvant when patients are at high risk for recurrence following curative-intent surgery and in the setting of metastatic disease.

Overall, the care of the patient with GIST remains in evolution. Surgery and TKI therapies remain critical for optimal chances for both cure and palliation. As well, limitations of current therapies persist. Management of wild-type tyrosine kinase patients remains a difficult subgroup (3, 4). As well, many non-exon III mutations continue to have increased risk of TKI failure (1, 2). More long-term, inability to tolerate TKI therapy remains a critical weakness in current therapies.

It is our hope and opinion that the articles presented in this series help define the current status of care for patients with GIST and allow an ever-increasing use of evidence-based approaches to optimize care, both hopefully maximizing cure and palliation as well as minimizing long-term complications these patients may suffer.

## Author contributions

All authors listed have made a substantial, direct, and intellectual contribution to the work and approved it for publication.
